# Rebamipide treatment ameliorates obesity phenotype by regulation of immune cells and adipocytes

**DOI:** 10.1371/journal.pone.0277692

**Published:** 2022-12-27

**Authors:** JooYeon Jhun, Jeonghyeon Moon, Se-Young Kim, Keun-Hyung Cho, Hyun Sik Na, JeongWon Choi, Yoon Ju Jung, Kyo Young Song, Jun-Ki Min, Mi-La Cho

**Affiliations:** 1 Rheumatism Research Center, Catholic Research Institute of Medical Science, College of Medicine, The Catholic University of Korea, Seoul, Korea; 2 Lab of Translational ImmunoMedicine, Catholic Research Institute of Medical Science, College of Medicine, The Catholic University of Korea, Seoul, Korea; 3 Department of Biomedicine & Health Sciences, College of Medicine, The Catholic University of Korea, Seoul, Korea; 4 Departments of Immunobiology and Neurology, Yale School of Medicine, New Haven, Connecticut, United States of America; 5 Division of Gastrointestinal Surgery, Department of Surgery, Yeouido St. Mary’s Hospital, Seoul, Korea; 6 Division of Gastrointestinal Surgery, Department of General Surgery, Seoul St. Mary’s Hospital, The Catholic University of Korea, Seoul, Korea; 7 Department of Internal Medicine, and the Clinical Medicine Research Institute of Bucheon St. Mary’s Hospital, Bucheon si, Gyeonggi-do, Republic of Korea; 8 Department of Medical Life Sciences, College of Medicine, The Catholic University of Korea, Seoul, Republic of Korea; The Chinese University of Hong Kong, HONG KONG

## Abstract

Obesity is a medical term used to describe an over-accumulation of adipose tissue. It causes abnormal physiological and pathological processes in the body. Obesity is associated with systemic inflammation and abnormalities in immune cell function. Rebamipide, an amino acid derivative of 2-(1H)-quinolinone, has been used as a therapeutic for the protection from mucosal damage. Our previous studies have demonstrated that rebamipide treatment regulates lipid metabolism and inflammation, leading to prevention of weight gain in high-fat diet mice. In this study, mice were put on a high calorie diet for 11 weeks while receiving injections of rebamipide. Rebamipide treatment reduced the body weight, liver weight and blood glucose levels compared to control mice and reduced both glucose and insulin resistance. Fat accumulation has been shown to cause pro-inflammatory activity in mice. Treatment with rebamipide decreased the prevalence of inflammatory cells such as Th2, Th17 and M1 macrophages and increased anti-inflammatory Treg and M2 macrophages in epididymal fat tissue. Additionally, rebamipide addition inhibited adipocyte differentiation in 3T3-L1 cell lines. Taken together, our study demonstrates that rebamipide treatment is a novel and effective method to prevent diet-induced obesity.

## Introduction

Obesity is a global health concern that increases the risk of various conditions such as cardiovascular diseases [1], type 2 diabetes [2], obstructive sleep apnea [3], depression [4], cancer [5], osteoarthritis [6] and asthma [7]. Like many other medical conditions, obesity is associated with many genetic and environmental factors including the gut microbiome [8–10]. Adipose tissue plays an active role in the regulation of energy metabolism and homeostasis through the expression and regulation of several regulatory genes such as peroxisome proliferator-activated receptor-γ (PPARγ) [11], lipoprotein lipase (LPL) [12] and adipocyte protein 2 (aP2) [13, 14].

The accumulation of visceral fat is associated with low-grade inflammation, which causes negative systemic changes over time [15, 16]. Adipose tissue produces and secretes adipokines and cytokines such as leptin [17], adiponectin [18], interleukin-4 (IL-4) [19], IL-6 [20], interferon-γ (IFN-γ) [21] and tumor necrosis factor-α (TNF-α) [22]. Pro-inflammatory cytokines promote the development of metabolic disease [23], type 2 diabetes and cardiovascular disease and aggravate pre-existing autoimmune diseases [24–26]. Obesity has been shown to increase IL-17-producing T cells in adipose and periphery tissues in human and a rodent model [27, 28]. Previous studies have shown that expansion of helper T (Th) 17 cells occurred in the murine obese model without modification of Th1 and Th2 cells [29]. These results suggest that obesity is closely related to increasing number of Th17 cells.

Oxidative stress can cause obesity by promoting the stimulation of white adipose tissue and increasing food intake by affecting hypothalamic neurons [30]. Oxidative stress plays a critical role in the development of obesity-related diseases through hyperglycemia [31], hyperlipidemia [32], mitochondrial dysfunction [33], impaired endothelial dysfunction [34], and chronic inflammation [35, 36]. Previous reports show that supplementation with antioxidants such as α-lipoic acid, catechins, and chlorogenic acids constituted an effective treatment against obesity [37–39]. Reactive oxygen species (ROS) play a critical role in Th cell differentiation [40, 41]. Elevated ROS induce inflammation through an increase of pro-inflammatory cytokines. Therefore, reducing ROS has therapeutic effects on various diseases.

Rebamipide is an amino acid derivative of 2-(1H)-quinolinone that plays a role in the synthesis of mucus glycoprotein, scavenging ROS, inhibition of neutrophil activation and suppression of inflammatory cytokines [42]. Rebamipide has previously been used as a therapeutic to prevent gastric damage caused by non-steroidal anti-inflammatory drugs (NSAIDs) by enhancing gastric mucosal protection and is under study for the treatment of rheumatoid arthritis, spondyloarthritis, osteoarthritis and dermatitis [43, 44]. The mechanism of action of rebamipide is inhibition of voltage-dependent L-type calcium channels, leading to a reduction of intracellular calcium, endoplasmic reticulum (ER) stress and mitochondrial dysfunction [45].

In this study, we confirm the regulatory and therapeutic effects of rebamipide on obesity in a high-fat diet induced obese mouse model. Mice received injections of rebamipide, then mouse spleenocytes were analyzed for immune cell population. Additionally, we examined the effect of rebamipide on adipocyte differentiation *in vitro*.

## Materials and methods

### Animals

Four-week-old C57BL/6 male mice (Orient Bio, Korea) (n = 10) were maintained under specific pathogen-free conditions. First, mice were fed a diet containing 60 Kcal of calories derived from fat in standard laboratory mouse chow (Ralston Purina, St. Louis, MO, USA) and water *ad libitum* for four weeks. Then, 100mg/kg rebamipide was administered by daily intraperitoneal injection after the mouse body weight reached 30g. All surgeries were performed under isoflurane anesthesia, and efforts were made to minimize suffering. The experimental protocol was approved by the Animal Research Ethics Committee at the Catholic University of Korea (ID number: 2017-0104-01) and all animals were treated and sacrificed in accordance with the guidelines of the Animal Research Ethics Committee of the Catholic University of Korea (permit number: CUMC-2016-0082-01), which conforms to the US National Institute of Health Guidelines. This study is performed in accordance with ARRIVE guidelines.

### Glucose and insulin tolerance tests

Mice were fasted overnight and subjected to intraperitoneal injection with glucose (1g/kg body weight). Blood glucose levels were measured with an Accu-Chek Performa (Roche, Basel, Switzerland) using whole blood taken from cut tail tips immediately before and 30, 60, 90, and 120 min after the injection of glucose (Sigma-Aldrich, St. Louis, MO, USA). The insulin tolerance test (ITT) was performed on nonfasted mice by intraperitoneal injection with insulin (1U/kg body weight) (Eli Lilly, Indianapolis, IN, USA). Blood glucose levels were measured before and 30, 60, 90, and 120 min after insulin injection, using an Accu-Chek Performa.

### Preparation of serum samples for biochemical analyses

Blood samples were collected in serum tubes from non-treated and rebamipide-treated mice at 14 weeks and stored at −70°C until use. The levels of total serum cholesterol were detected using commercial kits (Wako Co., Osaka, Japan). Aspartate aminotransferase (AST), alanine aminotransferase (ALT), glucose, free fatty acid, triglyceride, high-density lipoprotein (HDL)-cholesterol, and low-density lipoprotein (LDL)-cholesterol levels were measured using commercial kits from Asan Pharmaceutical Co. (Hwangseong-gi, Gyeonggi-do, Republic of Korea). Serum levels were detected by a Hitachi 7600 analyzer (Tokyo, Japan). To investigate the effect of fasting on blood glucose levels in mice, blood samples were obtained from the tails and glucose levels were measured using Accu-Chek (Roche) [46].

### Preparation of stromal vascular fraction cells

The epididymal fat pad (VAT) was carefully collected to exclude lymph nodes. Adipose tissue from each group of mice was enzymatically digested as described previously [47]. The stromal vascular fraction of the cell pellet was collected for Fluorescence-Activated Cell Sorting (FACS) analysis.

### Flow cytometry

To quantify the populations of Th1, Th2, Th17 and Foxp3-positive Treg cells, murine splenocytes and stromal vascular cells were stimulated with 25ng/mL phorbol myristate acetate (PMA) and 250ng/mL ionomycin (both from Sigma-Aldrich, St. Louis, MO, USA) and Golgi Stop (BD Biosciences, San Diego, CA, USA) in a 24-well plate and incubated for 4 hours. The stimulated splenocytes were stained with PerCP-conjugated anti-CD4 antibody (eBiosciences), then fixed and permeabilized using the Cytofix/Cytoperm Plus Kit (BD Biosciences) following the manufacturer’s protocol. Splenocytes were then reacted with fluorescein isothiocyanate (FITC)-conjugated anti-IL-17 antibody (eBiosciences). For analysis of the number of Treg cells, splenocytes were labeled with anti-CD4 and anti-CD25 antibodies, followed by fixation, permeabilization, and intracellular staining with anti-Foxp3 antibody as per the manufacturer’s instruction. To quantify the population of F4/80+CD11c+CD206- (M1) and F4/80+CD11c-CD206+ (M2) cells, mouse splenocytes were stained using allophycocyanin (APC)-conjugated anti-F4/80 antibody, FITC-conjugated anti-CD11c antibody and Phycoerythrin (PE)-conjugated anti-CD206 antibody (eBiosciences). All samples were analyzed by a FACS Calibur device (BD Pharmingen) [48].

### Differentiation of adipocytes

Murine 3T3-L1 pre-adipocytes were seeded at a density of 1.2 × 10^4^ and placed in a preadipocyte medium (Zenbio). Cells were grown to reach 100% confluency in a 24-well plate. The cells were incubated for 48 hours prior to initiating differentiation. After two days, confluent cells were replaced with an appropriate volume of 3T3-L1 differentiation medium (Zenbio), incubated for three days, then medium was replaced. 3T3-L1 adipocytes were pre-treated at 8 days, then dosed with 20μM, 100μM and 500μM of rebamipide every three days for 10 days. Some cells were used for mRNA extraction.

### Oil red O staining

Oil Red O staining of lipid droplets in differentiated adipocytes was performed as described previously. Day 14 3T3-L1 adipocytes were washed twice with cold PBS and then fixed in 10% formalin for 10 minutes at room temperature. Cells were washed again with PBS and incubated in 60% 2-propanol for 1 minute. Fixed cells were then placed in 0.18% Oil Red O solution for 15 minutes at room temperature. After staining, cells were washed with 60% 2-propanol with PBS. Sections were examined under a photomicroscope (Olympus, Tokyo, Japan). The stained cells were enumerated visually by four individuals, and the mean values were presented [49].

### Quantitative PCR

Messenger RNA (mRNA) was extracted using TRI Reagent (Molecular Research Center, Inc., Cincinnati, OH, USA) according to the manufacturer’s protocols. Complementary DNA (cDNA) was established using a SuperScript Reverse Transcription system (Takara Bio Inc., Kyoto, Japan). The cDNA was amplified with a Light-Cycler 2.0 instrument (software version 4.0; Roche Diagnostics, Mannheim, Germany). All reactions were conducted using Light Cycler FastStart DNA Master SYBR Green I mix (Takara), following manufacturer’s protocol [50]. The following primers were employed to amplify mouse genes: adiponectin (forward: GTCAGTGGATCTGACGACACCAA, reverse: ATGCCTGCCATCCAACCTG); leptin (forward: CCTCATCAAGACCATTGTCACC, reverse: TCTCCAGGTCATTGGCTATCTG); lipoprotein lipase (LPL) (forward: GGAAGAGATTTCTCAGACATCG, reverse: CTACAATGACATTGGAGTCAGG); adipocyte protein 2 (aP2) (forward: GATGCCTTTGTGGGAACCT, reverse: CTGTCGTCTGCGGTGATTT). All mRNA levels were normalized by the expression of β-actin.

### Statistical analysis

Data are exhibited as mean ± SDs of at least three independent experiments or at least three independent samples and with n = 10 mice in each group. *In vitro* experiments were independently performed three or more times and each experiment has at least three samples. One-way analysis of variance followed by Bonferroni’s *post hoc* test was used to compare differences between ≥ 3 groups. The Mann–Whitney *U* test was performed to compare numerical data between two groups. To assess the Gaussian distribution and the equality of variance, Shapiro–Wilk and Levene tests were used, respectively. *p* < 0.05 was considered statistically significant. Statistical analysis was performed using IBM SPSS Statistics 20 for Windows (IBM Corp., Armonk, New York, NY, USA) [51].

## Results

### Rebamipide reduces bodyweight in mice fed a high-fat diet

**Fig 1:**
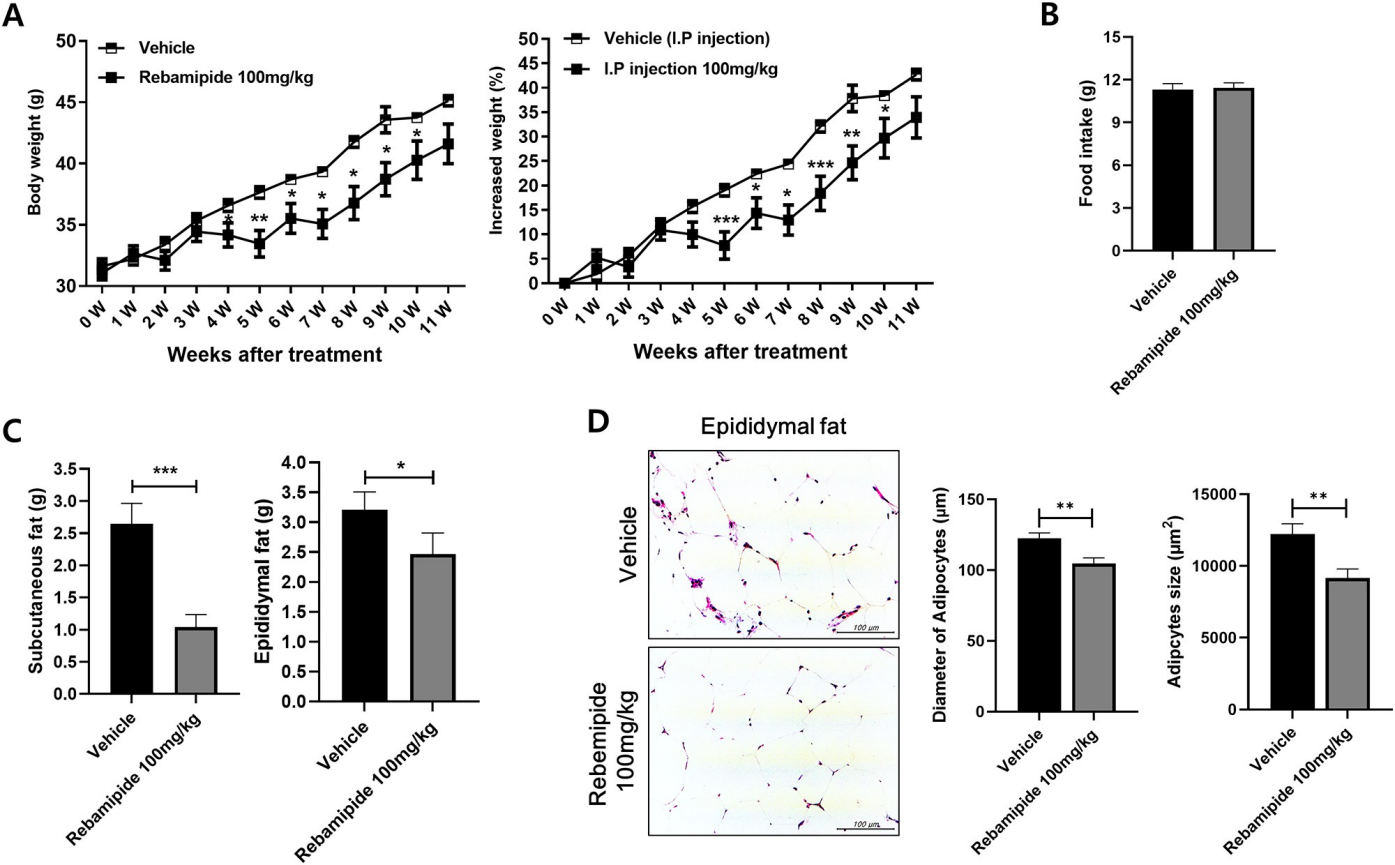
Protective effect against high-fat diet obesity of rebamipide. (A) Body weight and the ratio of increased weight of body weight were presented for 11 weeks. (B) Food intake in the rebamipide treatment group and vehicle group. (C) The subcutaneous fat and epididymal fat at the end of the experiment after 100mg/kg of rebamipide treatment. (D) H&E images of epididymal adipose tissues showed the crown-like structure, diameter, and size of adipocytes. *P < 0.05, **P < 0.01 and ***P < 0.005.

We investigated whether rebamipide would induce body weight loss in high-fat diet induced obese mice. The results show that intraperitoneal injection of 100mg/kg of rebamipide resulted in a decrease in body weight compared to vehicle controls. However, only the injection of 100mg/kg of rebamipide had a significant body weight change in mice (Fig 1A). Due to this, 100mg/kg of rebamipide was used in the following experiments. Administration of rebamipide did not affect food intake in mice (Fig 1B). At the end of the experiment, subcutaneous fat and epididymal fat weight of the rebamipide injected mice decreased (Fig 1C). In the H&E image of epididymal fat, crown-like structures were observed in mice fed a high-fat diet. However, in the adipose tissue of mice treated with rebamipide, crown-like structures were reduced, and it showed that the diameter and size of adipocytes decreased (Fig 1D). These results show that rebamipide inhibited the accumulation of fat in the body when the same caloric intake was consumed in the mouse condition with a high-fat diet.

### Rebamipide decreased the blood glucose level, insulin resistance and inflammation

**Fig 2:**
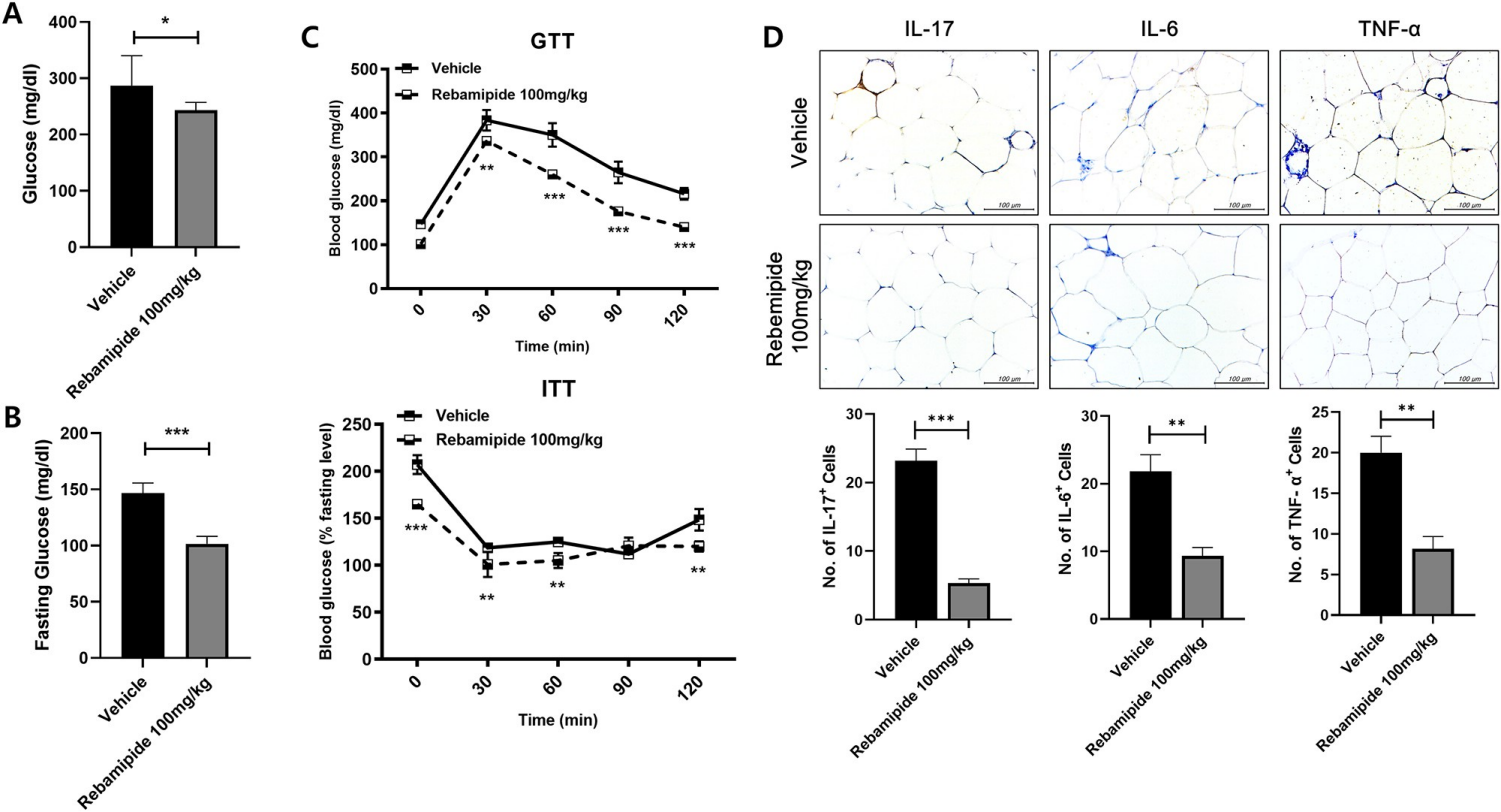
Rebamipide reduced blood glucose and inflammatory cytokines levels in mice. (A) Total blood glucose levels and (B) fasting glucose levels in treatment and control mice. (C) Glucose tolerance test (GTT) and insulin tolerance test (ITT) in fasted and non-fasted mice, respectively. (D) The immunohistochemistry images showed the expression of IL-17, IL-6 and TNF-α which were pro-inflammatory cytokines. *P < 0.05, **P < 0.01 and ***P < 0.005.

To measure blood glucose levels and insulin resistance, blood was collected at the end of the experiment and analyzed by a Glucose tolerance test (GTT) and insulin tolerance test (ITT). The total blood glucose level and fasting glucose level of mice was decreased after rebamipide injection (Fig 2A and 2B). Additionally, the increase and decrease of blood glucose levels was slower in rebamipide-injected mice, as compared to the vehicle. Baseline non-fasting blood glucose levels were notably lower in rebamipide-injected mice, as compared to the vehicle (Fig 2C). It is also known that fat accumulation not only raises blood glucose, but also raises the expression of pro-inflammatory cytokines. Therefore, we stained the representative pro-inflammatory cytokines IL-17, IL-6 and TNF-alpha through immunohistochemistry imaging in adipose tissue. As a result, rebamipide inhibited the production of pro-inflammatory cytokines in adipose tissue (Fig 2D). These results show that rebamipide had a hypoglycemic effect and anti-inflammatory effect in the high-fat diet mice.

### Liver steatosis was prevented by rebamipide

**Fig 3:**
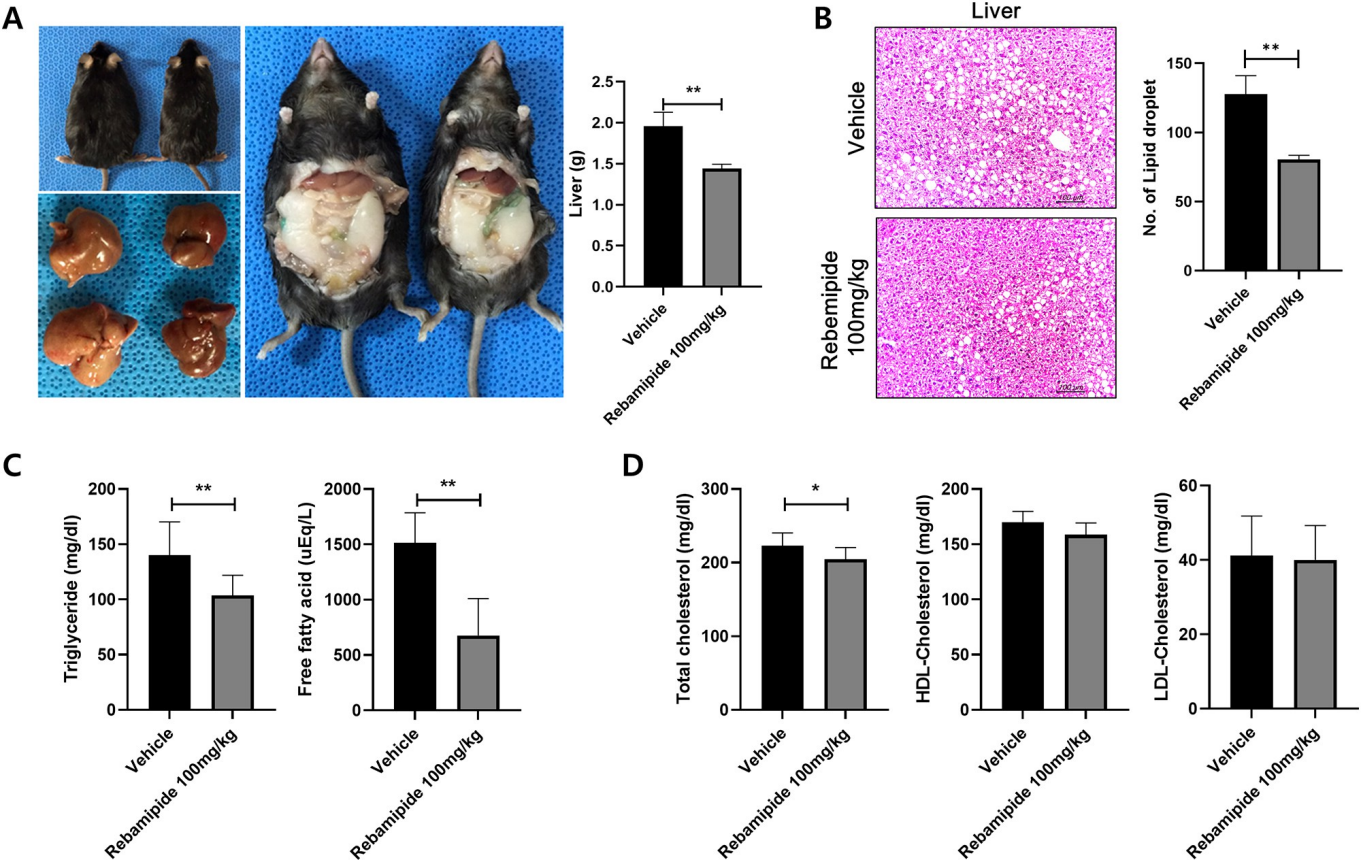
Rebamipide inhibits liver steatosis in mice. (A) Rebamipide decreased mouse body size, liver volume and liver weight. (B) The liver H&E images showed that rebamipide reduced the lpid droplet size in liver tissues. (C and D) The levels of triglyceride, free fatty acid, total cholesterol, high-density lipoprotein (HDL) and low-density lipoprotein (LDL) were monitored in the mice blood. *P < 0.05 and **P < 0.01.

We harvested the liver from endpoint mice to investigate the effect of rebamipide injection on liver steatosis in mice. Liver weight and volume were reduced by rebamipide treatment (Fig 3A). H&E images of liver tissue show that rebamipide inhibits lipid droplet accumulation in liver tissue (Fig 3B). We measured the levels of triglyceride and free fatty acid levels in blood, there were significantly reduced in rebamipide-treated mice (Fig 3C). The total cholesterol, high-density lipoprotein (HDL), low-density lipoprotein (LDL) were detected in blood. The total cholesterol level was notably decreased by rebamipide treatment. Although there was no significant difference in HDL and LDL-cholesterol levels, both tended to decrease in rebamipide-injected mice (Fig 3D). These data suggested that rebamipide has protective potential against obesity induced liver steatosis.

### Rebamipide regulated the population of helper T cells, regulatory T cells and macrophages

**Fig 4:**
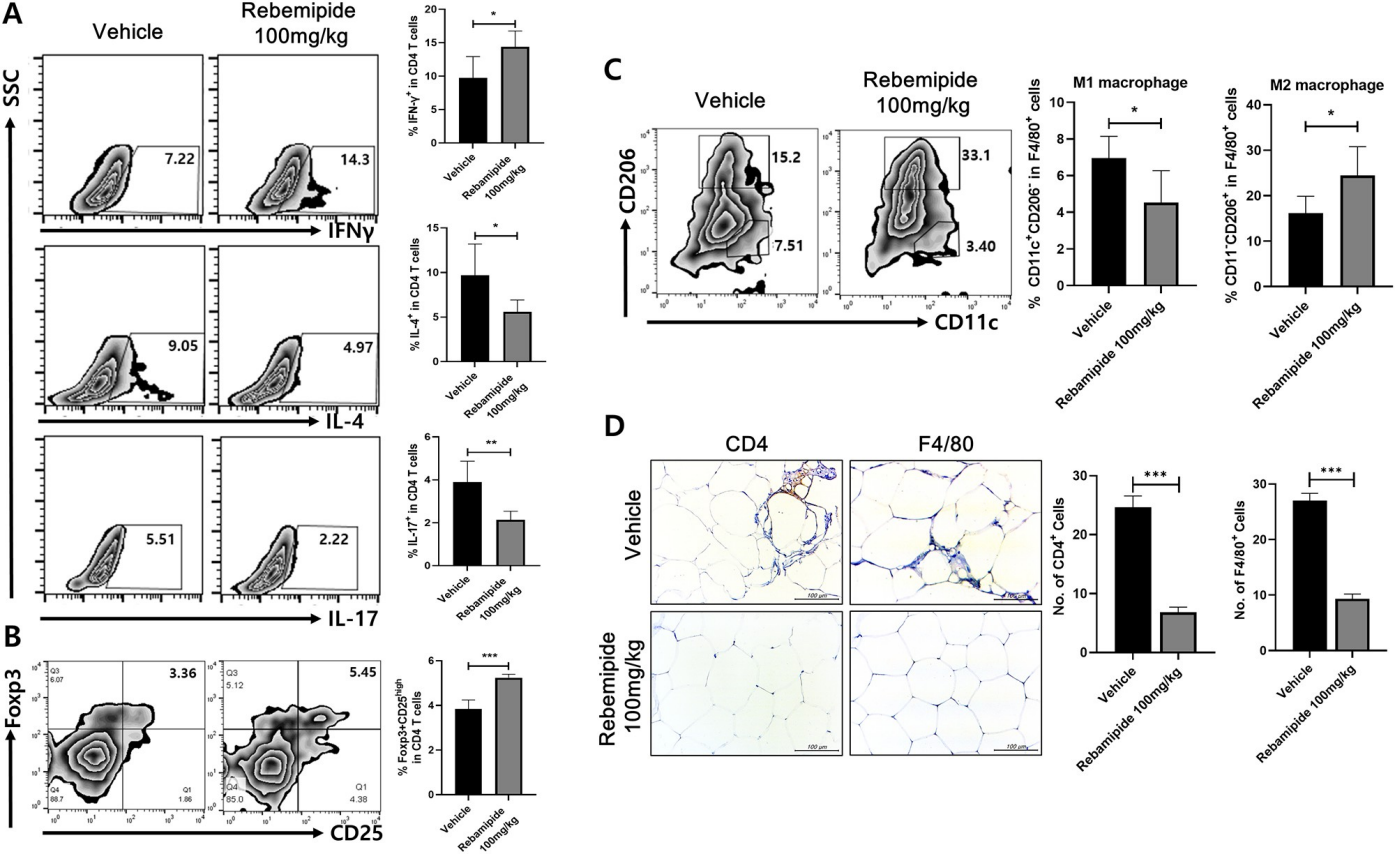
Rebamipide regulates the number of helper T cells (Th), regulatory T cells and macrophages in splenocytes and vascular fraction cells in adipose tissues of rebamipide-injected mice. (A) The population of helper T cells such as Th1, Th2 and Th17 was changed by rebamipide treatment in splenocytes. (B) The number of regulatory T cells was elevated in the rebamipide treated mice in epididymal tissues. (C) The numbers of macrophage 1 (M1) and macrophage 2 (M2) were changed by the treatment of rebamipide in epididymal tissues. (D) The immunohistochemistry images showed that rebamipide decreased the infiltrated CD4^+^ and F4/80^+^ cells in adipose tissues. *P < 0.05, **P < 0.01 and ***P < 0.005.

To determine whether rebamipide has anti-inflammatory functions, we performed flow cytometry on splenocytes, and stromal vascular fraction cells isolated from spleen tissue and the epididymal fat pad of mice. The population of helper T cells (Th) Th1 (CD4^+^ IFNγ^+^), Th2 (CD4^+^ IL-4^+^) and Th17 (CD4^+^ IL-17^+^) in splenocytes was affected by rebamipide. The number of Th1 cells increased, and the population of Th2 and Th17 cells were significantly decreased in rebamipide-injected mice (Fig 4A).

We further examined whether changes in Treg, M1 (F4/80^+^ CD11c^+^ CD206^-^) and M2 (F4/80^+^ CD11c^-^ CD206^+^) marker were observed in epididymal fat tissue. The population of regulatory T cells (Treg) was increased in rebamipide-injected mice (Fig 4B). The populations of macrophage 1 (M1) and macrophage 2 (M2) also had significant differences. M1, which has an inflammatory effect, was decreased while M2, which has an anti-inflammatory effect, was increased by rebamipide (Fig 4C). Immune cells infiltrated into adipose tissue were also monitored through immunohistochemistry staining. CD4^+^ cells and F4/80^+^ cells infiltrated into adipose tissue were reduced by rebamipide treatment (Fig 4D). These results demonstrate that rebamipide has an anti-inflammatory function via the regulation of immune cells and stromal vascular fraction cells.

### Adipocyte differentiation was inhibited by rebamipide

**Fig 5:**
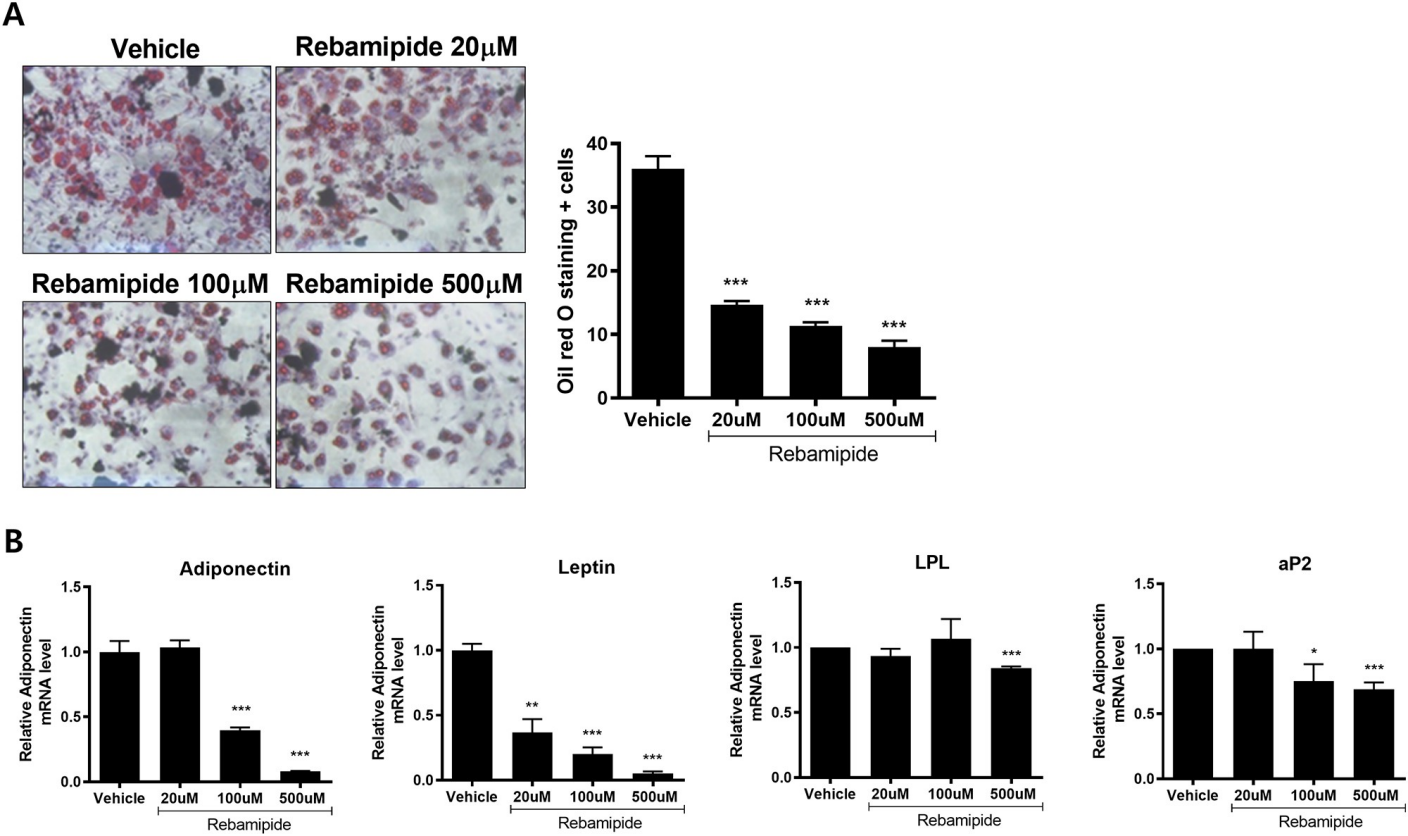
Rebamipide inhibits adipogenesis *in vitro*. (A) Adipocyte differentiation of 3T3-L1 was inhibited by rebamipide. (B) Expression of adipogenesis-related genes such as adiponectin, leptin, lipoprotein lipase (LPL) and adipocyte protein 2 (aP2) measured by qPCR. *P < 0.05, **P < 0.01 and ***P < 0.005.

To determine the effect of rebamipide on adipocyte differentiation, 3T3-L1 cells were treated with either 20μM, 100μM or 500μM of rebamipide. The numbers of Oil red O positive cells in the 3T3-L1 cells were reduced by all doses of rebamipide (Fig 5A). The quantitative PCR results showed the alteration of the expression of adipogenesis-related genes. Although the low dose of rebamipide only reduced leptin expression significantly, 100μM of rebamipide decreased the expression of adiponectin, leptin and adipocyte protein 2 (aP2). A dose of 500μM of rebamipide notably decreased the expression of adiponectin, leptin, lipoprotein lipase (LPL) and aP2. This result demonstrates that rebamipide has an inhibitory effect on adipocyte differentiation in 3T3-L1 cells (Fig 5B).

## Discussion

Obesity is a concerning medical condition caused by the abnormal accumulation of fat tissues [52]. Although the standard of obesity is different in each country, it negatively impacts the cardiovascular system [53], neuronal system [54], derma system [55], endocrine system [56] and immune system [57–59]. Various external conditions such as individual, socioeconomic, and environmental factors affect the prevalence of obesity, and many people find it difficult to improve [60–62]. Hence, there is a need for safe treatment against obesity.

Rebamipide is a medication used to increase mucosal protection against gastroduodenal ulcers [63]. Studies have shown that rebamipide has an anti-inflammatory effect through the inhibition of pro-inflammatory cytokines such as IL-1β and TNF-α [64, 65]. Previous work in our lab has demonstrated that rebamipide alleviates atherosclerosis via regulation of lipid metabolism and inflammation [46]. Also, rebamipide is reported to scavenge ROS and to show the protective effects on indomethacin-induced tissue peroxidations [66]. ROS is a key signaling molecule that play a critical role in the progression of inflammatory disorders [67]. ROS occurs the inflammation through inducing the DNA damage, lipid peroxidation, protein oxidative damage and NF-κB signaling pathway [67]. Here, we show that rebamipide has a prevention effect of body weight gain in high-fat diet mice.

Rebamipide injection in a high-fat diet induced obese mouse model regulated bodyweight, blood glucose level, liver weight, glucose tolerance and insulin tolerance compared to vehicle controls. Interestingly, food intake was not changed by rebamipide administration. Although not as risky as LDL and cholesterol, high blood triglyceride levels have been related to atherosclerosis, heart disease and stroke. Our study shows that rebamipide significantly decreased blood triglycerides, total cholesterol and free fatty acids in mice serum. These data show that rebamipide ameliorates not only body weight but also blood fatty acid level in *in vivo* mouse model.

Obesity has a strong association with immune cell abnormalities, and adipose tissue produces and releases various adipokines (leptin, adiponectin, resistin and visfatin) and pro-inflammatory cytokines (TNF-α, IL-4, IL-6) [68, 69]. This study confirmed that rebamipide has an anti-inflammatory effect on mice through a decrease in adipose tissue and pro-inflammatory cytokine production. Flow cytometry analysis of mice splenocyte and stromal vascular fraction cells showed a reduction in the number of Th2 and Th17 cells that can cause systemic inflammation and autoimmune diseases. Additionally, the population of Treg cells in splenocytes, which have an anti-inflammatory effect, was increased by the treatment of rebamipide. Further, rebamipide reduced the number of M1 macrophages, which have a pro-inflammatory affect and increased the population of M2 macrophages, which have an anti-inflammatory effect. These data show that rebamipide has an anti-inflammatory function through the regulation of immune cells and cytokine expression.

To investigate rebamipide inhibition of adipocyte differentiation, 3T3-L1 cells were treated with varying concentrations of rebamipide. This study confirmed the concentration-dependant inhibitory effect of rebamipide on adipocyte differentiation. Quantitative PCR data showed that rebamipide decreased adipocyte differentiation-related genes (adiponectin, leptin, LPL and aP2). In general, adiponectin has a role opposite to that of leptin and inhibits adipogenesis. However, adiponection is known to induce inflammation by increasing the expression of TNF-α. Rebamipide appears to have an anti-inflammatory effect by decreasing the expression of adiponectin [70].

The results of this study have limitations in that rebamipide does not show a clear mechanism for inhibiting fat accumulation. Further research needs to be conducted on this. However, Preliminary research results offer several possible mechanisms. Rebamipide acts to inhibit L-type voltage-dependent calcium channels [45]. Pancreatic beta cells regulate insulin secretion through L-type voltage-dependent calcium channels. In addition, the calcium channels such as Voltage-gated Calcium Channels (CaV), Transient receptor potential (TRP) and Calcium release activated channel (CRAC) increase the calcium levels in cytosol, which causes the expression of adipogenic genes such as PPARγ and C/EBPα [71]. A previous study has shown that antagonism against these calcium channels suppresses weight gain caused by high fat diets in mice [72]. Thus, rebamipide may play a role in inhibiting fat accumulation.

Taken together, the results of this study demonstrate a novel use for rebamipide as a treatment for fat reduction, adipocyte differentiation and the regulation of immune cells. However, further studies are needed to understand the precise mechanism of how rebamipide inhibits the accumulation of adipose tissue.
